# Efficacy of memory protocols in 9- to 89-year-olds’ memory for prior contacts

**DOI:** 10.1038/s41598-024-55267-3

**Published:** 2024-03-09

**Authors:** Deborah Goldfarb, Hana Chae, Haley R. Dawson, Jacqueline R. Evans, Ronald P. Fisher, Ariana Daneshbodi, Christian A. Meissner

**Affiliations:** 1 Psychology Department, Florida International University, Miami, USA; 2 Psychology Department, Iowa State University, Ames, USA

**Keywords:** Human behaviour, Epidemiology

## Abstract

Memory for prior contacts has several important applied implications, including contact tracing (for communicable diseases). Incomplete episodic memory reports, which occur across the developmental lifespan but are particularly relevant for children and older adults, may hamper such efforts. Prior research has shown that cognitively informed memory techniques may bolster recall of contacts in adults, but that work has not addressed the developmental efficacy of these techniques. Here we evaluated the effectiveness of such techniques for familiar and unfamiliar contacts within a sample of 9- to 89-year-olds in the context of an ongoing pandemic. The tested memory techniques bolstered recall across the lifespan, irrespective of whether the interview was conducted live with an interviewer or via a self-led interview. Children, emerging adults, and adults did not reveal any differences in memory productivity, however, older adults recalled fewer contacts. Implications for theory and application are discussed.

## Introduction

The COVID-19 pandemic laid bare the vital role that memory plays in public health efforts^1^. A key defense against the spread of infections is contact tracing, during which infected individuals recall those whom they may have infected via close contact, so those at risk can quarantine and prevent further infection. Recalling these prior contacts is an episodic memory task. Episodic memory reports, however, are often incomplete^2^ and the age of the individual recalling the contacts may further exacerbate this issue. Research generally reveals an inverse U-shaped relation between age and episodic memory performance, with individuals at the tail ends of the curve (children and older adults) performing worse than those in the middle^3,4^.

Cognitively informed interview techniques exist that can bolster memory for contacts. No study to our knowledge, however, has tested these protocols within a sample that included a full spectrum of ages from children to older adults. Further, no study has considered how cognitively informed interview techniques influence performance on recall for familiar and unfamiliar contacts. Evaluating memory for both familiar and unfamiliar contacts amid a pandemic permits a more externally valid assessment of this ability. The current study builds on the scant research assessing the efficacy of interview techniques that can bolster recall for contacts across the lifespan.

The inverse U-shaped relation between age and episodic memory performance may be explained by age-related differences in underlying cognitive mechanisms^3,4^. Some of these differences may be common to the two ends of the lifespan. For instance, both children’s and older adults’ reduced memory productivity may result from decreased working memory and processing speed abilities^3,5^. In contrast, some of these differences may be unique to children. For example, children’s smaller knowledge base regarding the topic to be recalled sometimes explains their decreased memory performance as compared to adults^6^.

To address potential age-related differences in episodic and autobiographical memory performance, researchers have developed interview protocols that leverage cognitively informed techniques for enhancing memory recall in both children and older adults^4,7,8^. One of the most effective protocols for eliciting information is the Cognitive Interview (CI)^9–11^. Critically, the CI bolsters memory performance, as compared to a standard interview, across the lifespan^4,10^. The CI increases the amount of information reported by using mnemonic principles (e.g., context reinstatement) to “cue” retrieval.

The CI’s use of context reinstatement and provision of additional cues (e.g., repeated and varied retrieval) during memory recall are thought to benefit individuals of all ages and, in prior research, have been shown to increase memory for contacts within a sample of adults^11,12^. Whether an identical CI-informed protocol could be used across the lifespan, versus using different protocols targeted for each age group, is an important question from both a theoretical and applied perspective. On a practical level, public health emergencies may limit the ability of agencies to administer protocols tailored to specific populations (e.g., children), making a one-size-fits-all protocol highly valuable. From a scientific perspective, using the same protocol across the lifespan allows direct comparisons of its effectiveness.

The efficacy of mnemonic principles across the lifespan may depend on how the interview is delivered. Interview studies have traditionally assessed interviewer-administered protocols. Given the resources required by such interviews, researchers developed a Self-Administered version of the CI (the “SAI”) that provides witnesses with written memory mnemonics and witnesses then write down what they recall, including descriptions of the persons that they saw^13^. Successful versions of the SAI have been created for child, adult, and older adult witnesses^14–17^. Prior research has found that an online self-led contact tracing interview (CTI) protocol was just as effective as an online (audio only) interview led by an interviewer to help adults recall contacts^12^.

Competing theories argue for and against the efficacy of a self-led interview for children and older adults. Self-led interviews may decrease requirements for processing speed and working memory by giving interviewees control over the interview pace (see Ref.^4^). Alternatively, the environmental support hypothesis^18^ posits that self-guided memory techniques challenge older adults. Similar patterns are seen with children, who often omit more information with open-ended questions than with closed-ended or forced-choice questions^19–21^ (but see Ref.^22^). Children’s low productivity may thus be explained by a need for additional guidance during retrieval, rather than a failure to encode. As such, it is unclear whether children and older adults might benefit more from a self-led or an interviewer-led interview.

Contact tracing efforts may also tap into developmentally salient memory topics as interviewees are asked to recall all contacts, including both those with whom they are familiar and unfamiliar. Recalling prior familiar contacts (vs. unfamiliar) may tap into unique developmental strengths. Unlike memory for strangers, memory for familiar others is based on repeated experiences, arguably creating an area of expertise. As expertise can help reduce developmental differences in memory performance^6,23,24^, children and older adults may show increased memory performance when describing familiar (vs. unfamiliar) individuals, potentially at levels equivalent to emerging adults and adults.

Yet, even when asked to recall familiar others, children may reveal developmental weaknesses. Studies involving live eyewitness interactions find that children generally report fewer details than adults when asked to describe others, with children and adolescents reporting only two or three details and adults reporting closer to seven or eight details^25–27^. Some studies even find that children fail to produce any person-description information when free recall questions are utilized^28^. These studies, however, focused primarily on describing unfamiliar others. Thus, it is unclear whether developmental differences will persist in an interview focused on eliciting information about both familiar and unfamiliar others.

### Present study

This study tested whether developmental differences persist when a Cognitively Informed Protocol (CogI) is utilized and if interview efficacy depends on delivery modality. To test these questions, a 2 (interview protocol: CogI vs. baseline) × 2 (modality: interviewer-led vs. self-led) × (age: 9 to 89 years) between-participants design was used, with age treated as both continuous and categorical in the analyses. We also considered the impact of contact familiarity on the role of age in recall productivity.

Data collection occurred during the COVID-19 pandemic, a theoretically important and externally valid environment in which to study memory for contacts, as contacts have a heightened saliency and centrality. The results of this study can inform our understanding of the efficacy of interview tools that could be used to respond to pandemics and other crises where the reporting of interactions with others is vital.

We predicted that episodic memory performance would reveal a U-shaped curve, with emerging adults and adults reporting more contacts than children and older adults. Further, we predicted that the CogI would produce more contacts than the baseline protocol, irrespective of interview modality. We expected participants to produce proportionally more familiar (vs. unfamiliar) contacts, and that developmental differences would only be evident for unfamiliar contacts. We also expected that the CogI would reduce developmental differences, particularly for unfamiliar contacts. We made no predictions regarding modality or any three- or four-way interactions.

## Methods

### Participants

Two hundred and sixty-eight participants, including children, emerging adults, adults, and older adults participated in the study (*M* = 36.89 years, *SD* = 23.74 years, range = 9 to 89 years). Approximately 24.3% of the sample were children (aged 9-17 years), 25.7% were young or emerging adults (aged 18–24 years), 25.0% were adults (aged 25–64 years), and 25.0% were older adults (aged 65-89 years). A power analysis, using a medium effect size^4,10^ and an alpha of 0.05 for a regression with 7 tested predictors, revealed that approximately 200 participants would yield power of at least 0.80. Additional participants were recruited due to potential attrition and to account for conditions outside of the researchers’ control.

Most of the sample identified as female (64.2%); the remainder identified as either male (34.3%) or nonbinary (1.5%). The sample identified as 6.3% Asian/Pacific Islander, 6.7% as Black, 9.3% as Hispanic or Latin, 63.4% as White, with the remaining 14.3% as either “other” or a multi-categorical background. The sample was a geographically diverse representation of locations within the United States and representation from states was spread across both age groups and interview conditions (See the supplementary table and figure for geographical distribution of participants in OSF at https://osf.io/235fq/?view_only=7d7673dea31447f383e6d3e20eb8ce23).

Data were collected from November 2020 to February 2021. This period was considered one of the peaks of the COVID-19 pandemic in the United States: The average number of new COVID-19 cases in the United States fluctuated from 70,000 to around 300,000 per day^29^. The vaccine was available to only some adults and was not available at all to those under the age of 18.

Very few of our participants had previously completed a CTI (7 participants or 2.6% of the sample). We asked participants to report their contacts over the previous six days. Approximately 13.9% of the sample went to school or their job exclusively in person (vs. remote) during that time while 11.4% percent reported going in person some days. Participants were outside of their homes an average of 3.94 hours (*SD* = 4.65 h) per day over the past six days. Around 10.8% of the sample reported primarily staying at home for the last six days; many of these people nonetheless reported contacts.

This study was approved by the relevant Institutional Review Boards. All research activities were performed in accordance with all relevant guidelines/regulations. Informed consent was obtained from all participants and/or their legal guardians.

### Measures

#### *Interview protocols*

Participants were randomly assigned to one of two interview conditions: Baseline interview protocol or CogI.

#### *Baseline interview protocol*

As the focus of the study involved recall of contacts, the baseline interview protocol was modeled after CTI protocols used in the ongoing COVID-19 pandemic^30,31^. Such interview protocols rely primarily on free recall and omit memory-enhancing mnemonics. Our baseline protocol mirrored those used in prior research^11,12^. Participants in the baseline condition received procedural instructions about what was referred to as the “SUPERTracer” interview protocol (e.g., explained the task, reassured them that we do not suspect that they have COVID-19, told them to access their calendar or phone for later use, and asked them to complete the interview in a quiet space), completed audio and video checks, and determined the relevant time interval covered by the interview (i.e., the past six days). In the first section of the interview, participants reported those with whom they lived during the past six days.

Next, “close contact” was defined for the participant using the relevant Center for Disease Control definition at that time: Being within six feet for a total of 15 minutes over the past 24 h, even if one of the parties was wearing a mask. Before continuing, participants successfully completed a comprehension check for the definition of a close contact. Participants received reminders of that definition throughout the interview.

Participants were then asked to report everyone with whom they had close contact over the defined time interval. Participants were then asked if they could recall any additional contacts from Work and School or Childcare. Participants were then given another opportunity to recall any additional close contacts. Finally, participants were encouraged to use their electronic devices, calendars, or other aids as prompts for any additional recall.

#### *CogI protocol*

The CogI began with instructions consistent with those in the baseline condition. The CogI additionally utilized social and cognitive memory techniques based on research assessing the efficacy of psychologically informed CTIs for adults^12^. Specifically, the interviewee received additional reporting instructions, including encouraging them not to guess but to report every detail. The CogI also included a “model statement” whereby the interviewer or the individual in the instructional video (depending on interview modality, as described in the following section) models the intense concentration and detailed reporting required. Participants were also encouraged to describe people as best they could, including details of what the contact looked like, the context of the encounter, or how they were related to the contact.

Next, an initial free recall of all contacts and events or activities over the relevant time interval was requested. Participants subsequently reviewed the recalled events one by one and reported any contacts from the event. For events where several people were present, participants were encouraged to mentally reinstate the context at the time the original event was encoded (e.g., what they were thinking, hearing, seeing, feeling) before reporting additional contacts. Participants were encouraged to close their eyes to help them focus.

Given the potential value of cues for improving memory performance across the lifespan, participants next heard five different categorical cues (i.e., Homes; Work and School or Childcare; People you do not know well; Social life; and Transportation) to facilitate recall of targeted contexts. Participants next heard a list of nine groups of specific cue words grouped into loose themes (e.g., repairs, helper, relatives, visitor, in-law, new, etc.) and were asked whether any further contacts were recalled for each group of cue words. Finally, as in the baseline condition, participants referred to their calendar, phone, or any other items that they believed would help them to remember their contacts^32^.

#### *Self-led conditions*

**Figure 1:**
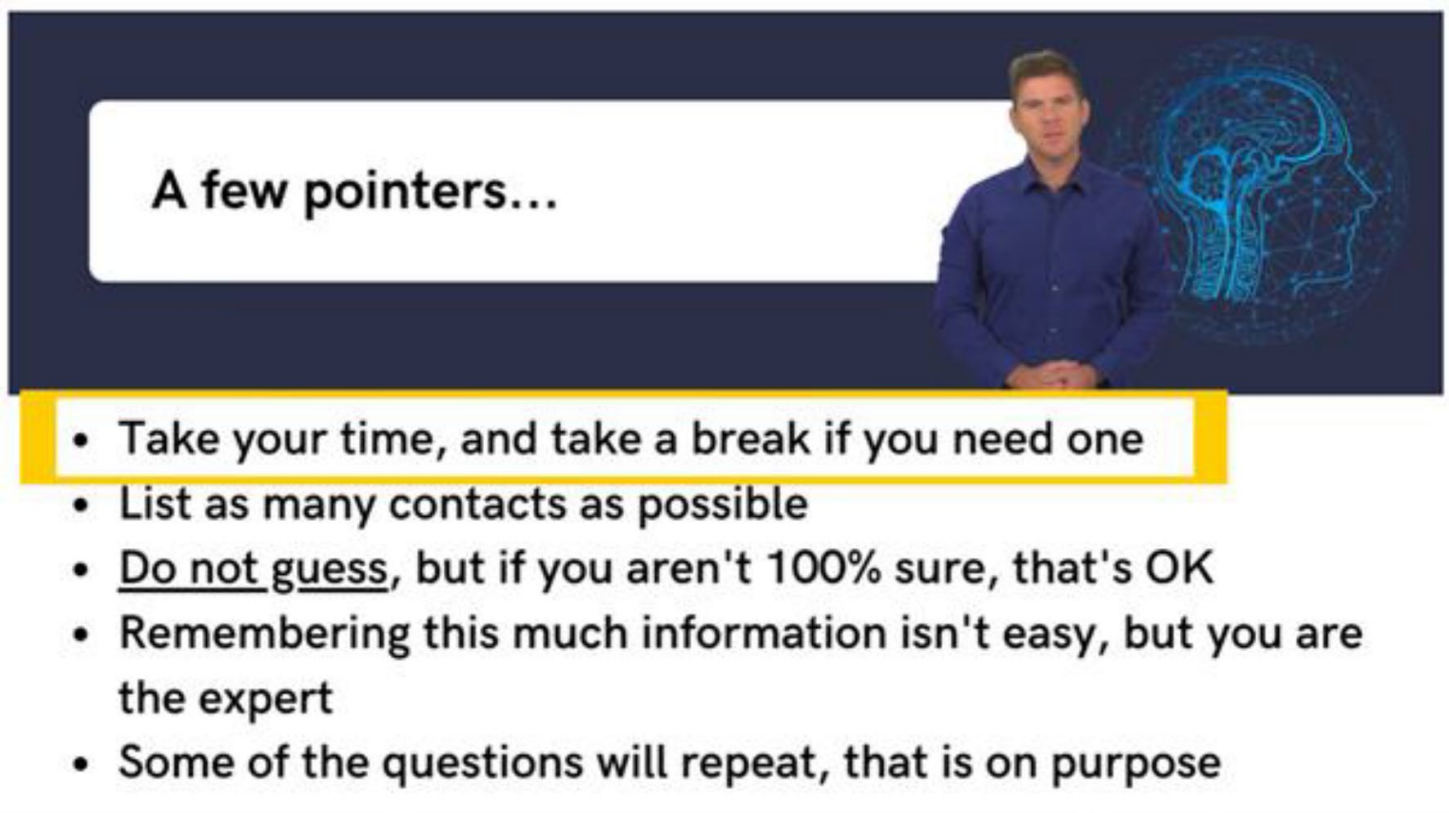
Sample Image of the Self-Led Video. *Note:* *Videos were in full color and narrated by a male presenter. The videos contained accompanying images and guided reading.*

In the Self-Led conditions, the protocol-specific instructions, cues, and prompts described above were delivered via a prerecorded video. The video was accompanied by visuals that provided instructional and pictorial aids for participants across the lifespan (see Fig. 1 for an example; Informed consent to publish identifiable image was obtained). Videos were pre-tested with around half a dozen individuals of different ages. Participants in the Self-Led conditions typed in their responses and did not interact directly with an interviewer.

#### *Contact details survey*

Participants provided information for each of the contacts that they recalled during the interview. To help ensure that any reported contacts were true contacts under the definition of the study, participants were given the opportunity to double-check whether a contact should be removed because the person was reported multiple times or was not a contact under the definition provided. For each contact, participants also reported whether they had their contact information, their confidence in whether this was a true contact, and the first day they had contact with this individual during the 6-day reporting period.

#### *Feedback survey*

Participants completed a feedback survey that gathered information on (a) participants’ current level of activities outside of the home (e.g., average number of hours outside of the home, if they go to work or school in-person, essential worker status), (b) participation in any prior contact tracing interview, and (c) feedback regarding participants’ interview experience. Many of these questions are outside of the purview of this article and are not discussed further.

### Procedure

Participants were recruited via several outreach methods, including online recruitment databases (e.g., Children Helping Science, Amazon’s Mechanical Turk), emails to community groups, social media advertisements, and a recruitment database of prior participants. Participants of all ages were recruited across different states to ensure our findings were not driven by locale. A live screening interview was conducted in which potential participants met with a researcher via Zoom to ensure they met the study’s eligibility requirements: They lived in the United States, were fluent in English, had a stable internet connection, and evidenced no hearing or processing limitations (parent-reported for child participants, self-reported for adults).

During this initial contact, necessary consents and assents were obtained, and participants completed a demographic survey followed by two working memory measures not reported here^4^. Eligible participants were then assigned randomly to an interview condition and scheduled for an interview appointment. Participants were not informed of the study’s purpose or their assigned condition to avoid increasing the salience or rehearsal of contacts. IRB approval was obtained from Florida International University.

On the scheduled interview date, participants in the Interviewer-Led Condition received a link to a secured video interview; those in the Self-Led Condition received a link to a self-administered Qualtrics survey containing the video prompts previously described. After completing the assigned interview protocol, participants completed the Contact Details and Feedback surveys.

### Coding

Our main dependent variable was the number of contacts reported by the participants. Two trained research assistants who were unaware of the hypotheses coded participants’ reports for the number of contacts. Coders counted something as a contact if the participant provided a name (either full name or first name), a label (e.g., “mom”), or described the contact (as descriptions could lead contact tracers to identifying further contacts (e.g., worker at Publix)). Contacts were counted only once (i.e., duplicates were removed).

The same coders also categorized the familiarity of each of the reported contacts as either: A family member or a non-family roommate (highly familiar); a friend, teacher, classmate, or colleague (somewhat familiar); or a stranger or other unfamiliar person (unfamiliar). Where familiarity of the contact could not be coded, the contact was coded as undefinable. Each contact was coded only into one of the four categories. After establishing high reliability (*k* = 0.923) between two coders on 18% of the participants (i.e., 48), the remaining participants were divided between the two coders for familiarity coding.

## Results

### Preliminary analysis

Given the unprecedented nature of the time during which the data were collected, we conducted preliminary analyses to understand participants’ exposure to contacts during this phase of the pandemic. Contact counts varied between 0 contacts (*n* = 3) and 61 contacts (*M* = 9.13, *SD* = 8.73). Given the considerable variance in contacts, we excluded from the analyses any participants (*n* = 9) who had total contacts that were three standard deviations greater than the average (> 35 contacts).

We first conducted curve estimation analyses to determine whether the total number of contacts, measured continuously, reported by participants revealed the typical developmental U-shaped curve. Contrary to our initial predictions, curve estimation did not reveal a significant quadratic curve (*b* = 0.001, *F* = 7.28, *p* = 0.537). Instead, both a negative linear (*b* = − 06, *F* = 14.21, *p* < 0.001) and a negative logarithmic curve showed excellent fit (*b* = − 1.96, *F* = 13.78, *p* < 0.001). To test whether there was an increase in performance with age amongst the children, a one-way analysis of variance (ANOVA) comparing the number of contacts recalled between children ages 9-11, 12-14, and 15-17 was conducted; it was also not significant (*F* = 0.121, *p* = 0.886). Given the similar fit for both curves and that the negative logarithmic curve revealed a pattern contrary to prior hypotheses (increased performance at the youngest ages and stability at the remaining ages) and the results of the ANOVA analyses, we maintained a linear curve for our analyses.

We next analyzed whether there were age differences in the amount of time that participants spent outside of the house, as such differences could explain any age differences in the number of contacts reported (e.g., older adults staying home more). Correlational analyses revealed that age in years negatively predicted the average daily amount of time spent outside of the house over the past six days (*r* = − 0.16, *p* = 0.009). To ensure that any age difference effects were not driven by such differences, we controlled for average hours spent outside of the house in the analyses below. All analyses were conducted using SPSS.

### Effects of interview protocol, modality, and continuous age on number of contacts recalled

To test the effect of age and condition on the number of contacts recalled, we ran linear regression analyses with the number of hours spent outside of the home in the first model, age in the second model, interview protocol and modality in the third model, the two-way interactions in the fourth model, and the three-way interaction in the fifth and final model. Both age and interview protocol were significant predictors (*b* = − 0.041, *t* = − 2.88, *p* = 0.004 and *b* = 2.32, *t* = 3.50, *p* = 0.001), with younger age and the CogI condition significantly predicting recalling more close contacts. Modality also significantly interacted with age, *b* = − 0.058, *t* = − 2.10, *p* = 0.037. This effect is described more below. No other predictors were significant.

### Effects of interview protocol, modality, and binned age on number of contacts recalled

Given the ongoing and differential impact of the COVID-19 pandemic across age groups at the time that the data were collected (e.g., the increased mortality rates for older adults^33^; return for in-person schooling for many children and emerging adults) and to further probe the linear effects found above, we conducted additional analyses utilizing ANOVAs after binning age into four groups: Children (9- to 17-years-old), emerging adults (18- to 25-years-old), adults (26- to 64-years-old), and older adults (65-years-old and above). Specifically, we tested the effects of interview protocol, modality, and age on the total number of contacts recalled.

A 2 (interview protocol: CogI, baseline) × 2 (modality: interviewer-led, self-led) × 4 (age: children, emerging adults, adults, and older adults) univariate analysis of covariance (ANCOVA) with average number of hours spent outside of the home as a covariate paralleled all of the results above, except for one. There were main effects for age (*F*(3, 255) = 3.49, *p* = 0.016, *η *_p_^2^ = 0.04) and interview protocol (*F*(1, 255) = 12.51, *p* < 0.001, *η*_p_^2^ = 0.05). Neither the main effect of modality (*F*(1, 255) = 0.15, *p* = 0.695) nor the Interview Protocol x Modality interaction was significant (*F*(1, 255) =1.04, *p* = 0.308). Despite our predictions otherwise, age did not significantly interact with protocol (*F*(3, 255) = 0.33, *p* = 0.805). The exploratory interaction of modality and age, unlike with the previous analysis, was not significant (*F*(3, 255) = 2.06, *p* = 0.107). Similarly, the exploratory three-way interaction of interview protocol, modality, and age was not significant (*F*(3, 255) = 0.20, *p* = 0.895).

**Figure 2:**
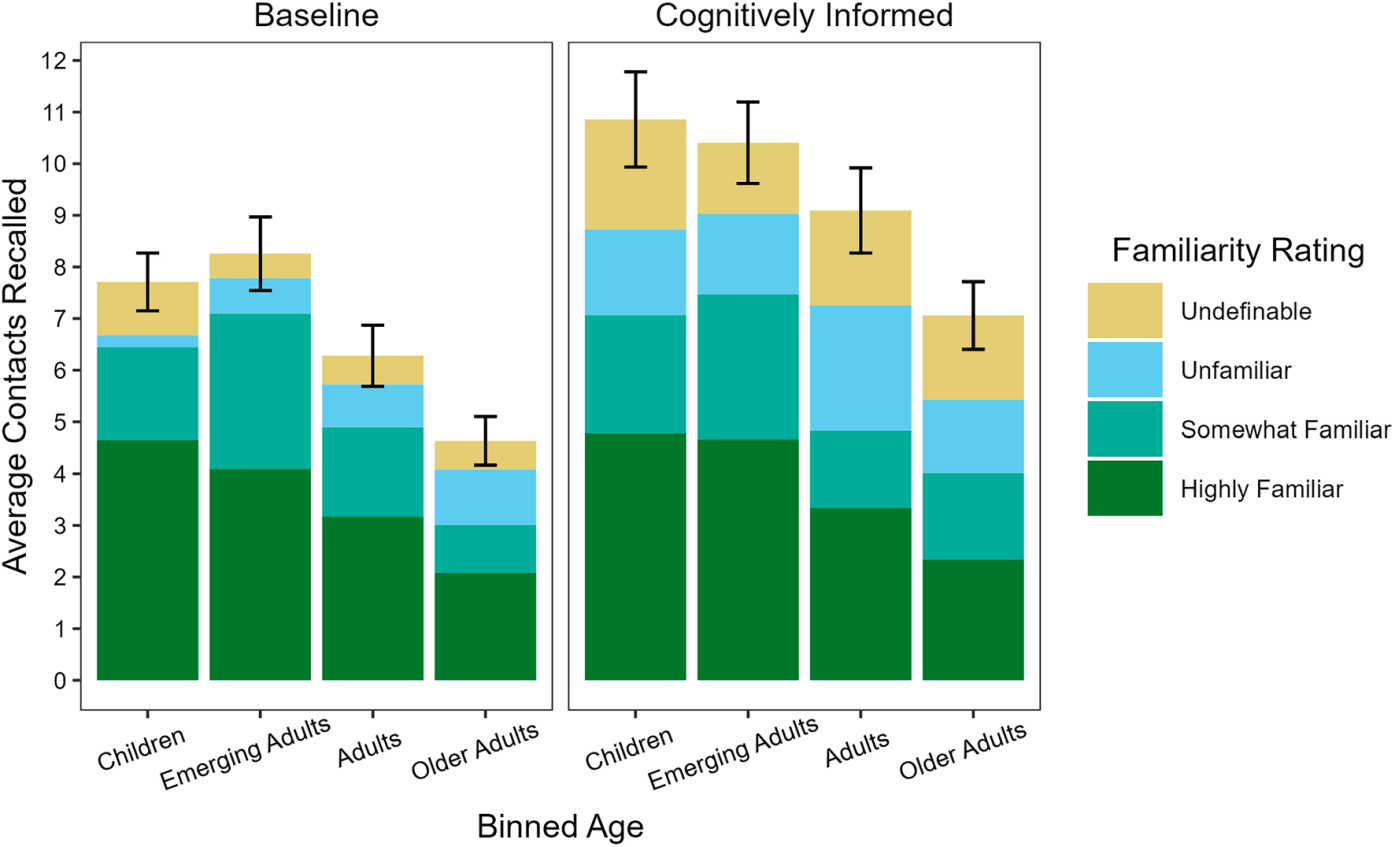
Familiarity of Contacts Recalled Across Age and Interview Protocol. *Note: Given that there was no significant difference between the number of contacts reported by participants in the self-led condition* (*M* *= 7.99*, *SD = 5.20) and the interviewer-led* *condition* (*M = 8.00, SD* *= 6.72*) *the figure is collapsed across the modality condition. For completeness, undefinable contacts were included in the figures and calculations, though familiarity information was unable to be determined for these contacts. Error bars shown are Confidence Intervals.*

As anticipated, participants in the CogI condition recalled more contacts (*M* = 9.19, 95% CI [8.25, 10.13]) than participants in the baseline condition (*M* = 6.83, 95% CI [5.91, 7.74]). Although the interaction of interview protocol and modality was not significant, means are shown below for illustrative purposes (Fig. 2). Despite predictions otherwise, pairwise comparisons revealed that older adults recalled fewer contacts than both children and emerging adults (*p*s = 0.006; Fig. 2). Older adults did not differ from adults (*p* = 0.143). Children, emerging adults, and adults did not differ from each other (*p*s > 0.175).

Of note, the modality and age interaction found in the linear regressions did not replicate in this analysis. Post-hoc comparisons, however, revealed that older adults recalled fewer contacts when questioned by an interviewer (*M* = 4.89, 95% CI [3.03, 6.75]) than when they completed a self-led interview (*M* = 7.81, 95% CI [6.01, 9.62]) but such differences were not seen in the other age groups.

### Impact of familiarity on developmental memory findings

We next analyzed the impact of contact familiarity on recall productivity. We calculated the proportion of each participant’s recalled contacts that were either highly familiar, somewhat familiar, or unfamiliar and ran a repeated-measures ANCOVA with interview protocol, modality, and binned age as between-subjects variables and proportion familiarity as the within-subjects factor. As the focus of this analysis is differences in familiarity, only the within-subjects effects and contrasts are discussed. This analysis resulted in a main effect for familiarity (*F*(2, 472) = 43.63, *p* < 0.001, *η*_*p*_^2^= 0.16), which was qualified by several interactions: Protocol X Familiarity, *F*(2, 472) = 5.13, *p* = 0.006, *η*_*p*_^2^= 0.02, Age X Familiarity, *F*(6, 472) = 4.53, *p* < 0.001, *η*_*p*_^2^ = 0.05, and Age X Modality X Familiarity, *F*(6, 472) = 2.57, *p* = 0.019, *η*_*p*_^2^= 0.03.

Overall, participants reported a greater proportion of highly familiar contacts (*M* = 0.45, *SD* = 0.34) than they did somewhat familiar (*M* = 0.25, *SD* = 0.29; *p* < 0.001) and unfamiliar contacts (*M* = 0.21, *SD* = 0.26; *p* < 0.001); there were no differences between somewhat familiar and unfamiliar contacts (*p* = 0.191). Familiarity interacted with interview protocol (Fig. 2) such that the CogI condition yielded more unfamiliar contacts (*M* = 0.25, *SD* = 0.26; *p* = 0.016) than the baseline condition (*M* = 0.17, *SD* = 0.25), while the baseline condition yielded a greater proportion of highly familiar contacts (*M* = 0.51, *SD* = 0.36; *p* = 0.007) than the CogI condition (*M* = 0.39, *SD* = 0.31). The two protocols did not differ as to the proportion of somewhat familiar contacts (Baseline: *M* = 0.27 *SD* = 0.31; CogI: *M* = 0.22, *SD* = 0.26; *p* = 0.113).

The familiarity effect was also subsumed by the separate three-way interaction with age and modality. Pairwise comparisons with an LSD correction revealed that older adults recalled (a) a greater proportion of highly familiar contacts (*M* = 0.49, *SD* = 0.35; *p* = 0.016) and (b) a smaller proportion of somewhat familiar contacts (*M* = 0.13, *SD* = 0.22; *p* = 0.010) in the interviewer-led interview than they did in the self-led interview (*M*s = 0.30 and 0.30, *SD*s = 0.31 and 0.32 respectively). Adults, emerging adults, and children did not reveal any differences in the proportion of familiar contacts at any level of familiarity between either the interviewer-led interview or the self-led interview.

In considering the interaction from a developmental perspective, in the self-led condition, children, emerging adults, and adults also all remembered a greater proportion of highly familiar contacts than did older adults (*p*s < 0.006; Fig. 2). In contrast, adults recalled fewer somewhat familiar contacts than older adults (*p* = 0.029). Children and emerging adults reported fewer unfamiliar contacts than older adults (*p*s < 0.005) and adults (*p*s < 0.035). Turning to the interviewer-led condition, there were no significant differences as to highly familiar contacts. Older adults recalled proportionally fewer somewhat familiar contacts than emerging adults (*p* = 0.010) and children and emerging adults recalled fewer unfamiliar contacts than adults and older adults (*p*s < 0.035).

## Discussion

We sought to expand our understanding of the development of memory productivity by recruiting a diverse sample ranging from 9 to 89 years of age. As participants of all ages engaged in the same memory task, the results provide unique insights into the role of age plays in both memory for contacts and the efficacy of scientifically-informed memory techniques. Recall was bolstered for participants of all ages using such techniques, with the scientifically-informed protocol being particularly powerful at helping recall of unfamiliar contacts. Irrespective of the interview protocol used, there was a decreasing linear effect of age. Categorical analyses, however, revealed that children were similarly productive in remembering prior contacts as emerging adults and adults. Older adults, however, recalled fewer contacts compared to children and emerging adults. These findings are partially explained by the familiarity of the information to be remembered. Findings are discussed below, along with application to contexts within which memory for contacts is vitally important, including public health crises like the COVID-19 pandemic.

Our scientifically-informed interview protocol increased the number of contacts recalled for participants of all ages. This finding held even after controlling for the time that participants reported spending outside of their homes. Although individual age-cohort-based studies demonstrate the efficacy of these techniques^4,10,12^, this study is the first to our knowledge to do so in one study using the same techniques across the lifespan and analyzing the effect of age. Of note, the benefit of the scientifically-informed interview protocol was particularly seen in the recall of unfamiliar contacts, which aligns with the belief that recall of these contacts may require the greatest mnemonic assistance.

Our protocol’s effects held even when the interview was self-led, replicating prior work^12^. Indeed, post-hoc ANOVA analyses showed that older adults produced more contacts with a self-led (vs. interviewer-led) interview. This finding runs contrary to the environmental support hypothesis^18^, which suggests that older adults would struggle with self-guided techniques. As our interviewer-led interviews were conducted via video conference (rather than in-person), it will be important for future studies to assess whether the modality of interaction (online vs. in-person) might qualify the efficacy of self-led interviews for older adults. While children perform similarly across online and in-person laboratory tasks^34^, video interviews may have introduced additional technical, auditory, or other processing challenges for our older adults. The pre-recorded instructions in the self-led context allowed for the pausing or replaying of instructions, potentially helping support decreased working memory capacity. We are limited in our ability to analyze such use of the videos here, and thus additional research is needed for this finding.

There are numerous applied implications for these findings. CTIs, where an infected individual remembers contacts who they may have infected (so those contacts may be encouraged to self-quarantine), are necessary to recover from pandemics^35^. Search and rescue recovery efforts after a natural disaster or a mass shooting event also often rely on survivors’ memory of who was or may still be in a location. Here, we extend prior work showing that the efficacy of interviews focused on recalling other people can be bolstered utilizing scientifically-informed memory techniques^11,12^. Such techniques can be used across the lifespan, including within a self-led interview format that can save a sizable number of person-hours.

Analyses considering the impact of age on memory revealed an asymptotic trend (rather than the conventional U-shaped trend). Specifically, children, emerging adults, and adults did not differ in recall productivity. Children and emerging adults did, however, recall more contacts than older adults. There are a few potential explanations for this counterintuitive finding.

First, roughly half of the contacts recalled were highly familiar contacts. Developmental differences in memory performance often dissipate, or disappear, when the target material is familiar to children and adolescents^6,36^. Developmental strengths in younger ages in the current study may thus be driven by the familiar nature of the information to be recalled. Indeed, both children and emerging adults recalled proportionally more familiar contacts than their older counterparts. Additional research is also necessary to understand whether this finding is driven by the larger social networks inherent in this developmental phase, as compared to older adults^37^, the result of developmental differences in relying on schematic memory rather than episodic memories, the use of electronic devices or other mnemonic cues that may have disproportionately benefited younger individuals recall, or true differences in productivity.

Second, the developmental findings may be an artifact of collecting data during a pandemic. Social distancing decreases interpersonal interactions, yet likely increases their salience, given the concomitant risk of infection. This may have increased the memorability of close contacts for participants of all ages, but particularly for children and emerging adults for whom social networks, particularly peers and family members, are of vital importance^38,39^. Further, given that earlier variants of the COVID-19 virus disproportionately impacted older individuals, older adults likely further tightened their social networks resulting in fewer contacts to recall. Although our results held after controlling for the number of hours spent outside of the home, we did not capture the contexts within which those hours were spent (e.g., school versus shopping versus sitting in an empty office), and additional research is necessary here.

Third, we may not have found age differences here, even when we compared the number of contacts recalled just within the sample of children, given that our youngest participants in the study were nine. Although memory development continues throughout childhood and early adolescence, even our youngest participants were old enough to have developed several relevant cognitive skills. For instance, the ability to bind items (here contacts) to time (here the six-day recall period) develops throughout middle childhood and reaches stability around 11 years of age^40^. Children younger than nine would thus likely struggle to remember information in such a paradigm given their more limited recall abilities. We are unable to draw such conclusions here.

### Limitations

A significant limitation of this study is that we are hampered here in our ability to measure the accuracy of the close contacts reported as we do not have records of participants’ actual contacts during the relevant period. Tracking such interactions is a challenging endeavor for all studies given the associated costs, but such challenges are heightened for a national study during an ongoing pandemic. Participants, particularly child participants who have increased vulnerabilities for suggestibility, may have thus inadvertently padded their reported contacts with schematic memories. Further, participants knew that they were participating in a research study and not an actual public health interview, which may have shifted their motivations to report (or not to report). All participants here also volunteered and chose to take part in research, unlike real contact tracing interviews where individuals are often cold-called as the result of a positive test result. Participants were also subject to different state requirements regarding social distancing, which may have created different incentives to report contacts. Cognitively-informed interviews, like the CogI, are, on average twice as long as standardized interviews, like the baseline interview^12^; the impact of the increased length on whether participants are willing to engage in such interviews must be further studied. Finally, our live interviewers were trained research assistants and not professional contact tracers, who devote their lives to improving public health.

## Conclusion

In addition to the theoretical value of the data highlighted above, the COVID-19 pandemic heightened the applied value of research considering both developmental differences in the ability to recall familiar and unfamiliar contacts as well as the efficacy of interview protocols across the lifespan. The knowledge gained here directly informs our ability to conduct productive interviews with individuals of all ages regarding past contacts. It also builds upon the continually expanding body of research regarding the contexts under which age may matter for memory^3^ and highlights the value of children’s memories in recalling salient information and events.

## Data Availability

The data that support the findings of this study, the syntax, and the materials are openly available in OSF at https://osf.io/235fq/?view_only=7d7673dea31447f383e6d3e20eb8ce23 and the analyses were pre-registered aspredicted.org. Changes to pre-registered analyses are noted above.
